# The impact of regional deprivation on stroke incidence, treatment, and mortality in Germany

**DOI:** 10.1186/s42466-023-00232-0

**Published:** 2023-02-09

**Authors:** Matthias Hans Belau, Heiko Becher, Maya Riefflin, Dirk Bartig, Lars Schwettmann, Christopher Jan Schwarzbach, Armin Grau

**Affiliations:** 1 Institute of Medical Biometry and Epidemiology, University Medical Centre Hamburg-Eppendorf, Martinistraße 52, 20246 Hamburg, Germany; 2 Heidelberg University Hospital, Heidelberg Institute of Global Health, Heidelberg, Germany; 3 Department of Neurology, St. Josef Hospital Bochum, Ruhr University Bochum, Bochum, Germany; 4 Department of Health Services Research, School of Medicine and Health Sciences, Carl von Ossietzky University of Oldenburg, Oldenburg, Germany; 5 Department of Neurology, Hospital of the City Ludwigshafen, Ludwigshafen, Germany

**Keywords:** Deprivation, Stroke incidence, Stroke mortality, Stroke treatment, Germany

## Abstract

**Background:**

Regional deprivation has been shown to be an influential factor in stroke incidence risk. However, there is a paucity of knowledge on regional differences in stroke incidence and mortality in Germany.

**Methods:**

We assessed data from the Diagnosis Related Groups statistics (2016–2019) and the German Federal Registry of Physicians (2019). Negative binomial regression analysis was used to examine the association between the German Index of Multiple Deprivation 2015 covering 401 districts and district-free cities in Germany and stroke incidence, treatment, and mortality.

**Results:**

The adjusted rate ratios of stroke incidence and mortality with the highest deprivation level compared with the least deprived area were 1.161 (95% CI [1.143, 1.179]) and 1.193 (95% CI [1.148, 1.239]), respectively. Moreover, this study revealed that physician density was higher in district-free cities compared to districts.

**Conclusions:**

Our results indicate that regional deprivation is associated with incident and mortality cases of stroke, necessitating a more targeted approach to stroke prevention in deprived regions.

**Supplementary Information:**

The online version contains supplementary material available at 10.1186/s42466-023-00232-0.

## Background

Stroke is the second-leading cause of death worldwide and socioeconomic inequalities in stroke morbidity and mortality are widening [1]. In Germany, stroke is common [2] and associated with a high economic burden [3]. Adverse socioeconomic conditions at the level of individuals and the level of residential areas have been posited to contribute to the development of stroke [4]. Individual socioeconomic status (SES) comprises factors such as education, income, and occupation, and childhood SES is a well-known risk factor for lifetime stroke [5–7]. Area-based socioeconomic measures in form of deprivation indices, however, focus more on the area-level indicators, such as the unemployment rate [8].

Several studies show geographical variations together with social deprivation in stroke incidence risk [9–12]. Some studies indicate that area-based deprivation is associated with a poorer quality of stroke care and treatment [13, 14]. A few studies report associations between social or socioeconomic deprivation and stroke mortality [15, 16]; however, studies examining the impact of area-based deprivation on stroke mortality in Germany are scarce.

Germany is divided into administrative divisions at different spatial levels, with the federal states being at the highest level and the municipalities at the lowest. However, there is a paucity of knowledge on spatial-structural differences in stroke incidence and mortality. One study revealed a relationship between regional deprivation and the occurrence of acute ischemic stroke in the German state of Rhineland-Palatine [10]. Another study examined time trends and differences in stroke mortality between East and West Germany and found that the differences were marginal [17]. To our knowledge, no study so far has examined the impact of area-based deprivation on stroke incidence and mortality at the spatial level of districts in all of Germany. Information on socioeconomic inequalities in stroke incidence and mortality at the district level could be useful to tailor health promotion, prevention, and treatment strategies in regions with high levels of socioeconomic deprivation.

Thus, we aimed to investigate the association between regional deprivation and stroke incidence and mortality, considering all documented hospitalized cases with the main diagnosis of ischemic stroke, and the effect of regional deprivation on key elements of stroke care and treatment. It was hypothesized that the population in regions characterized by high levels of deprivation has significantly higher rates of stroke hospitalization incidence and mortality and poorer rates of stroke care and treatment.

## Methods

### Data sources and measurement

We assessed data from the Diagnosis Related Groups (DRG) statistics [18] of all hospitalized patients resident in Germany who were assigned the main diagnosis of the ICD-10-GM (International Statistical Classification of Diseases, German Modification) code I63 for ischemic stroke in the period from 2016 to 2019. To avoid multiple documentation, patients with "discharge code 06" (transfer to another hospital) were excluded, and for patients admitted to more than one hospital, only the first hospital stay was counted. Code I63 includes patients with first or recurrent ischemic stroke of any cause. In-hospital strokes were coded as secondary diagnoses and were not included in the analysis. Information on cases of incident stroke was available at the district level and stratified by 5-year age group and sex. Deaths due to stroke refer to patients with "discharge code 07" (death).

Regional deprivation was assessed using the German Index of Multiple Deprivation from 2015 (GIMD 2015). The GIMD 2015 has been established based on the method used by Maier et al. [19, 20], and developed by Noble et al. [21]. It considers 7 different domains with different weighting (%) [22]: income (25%), employment (25%), education (15%), district revenue (15%), social capital (10%), environment (5%), and security (5%). The GIMD 2015 was calculated at the spatial level of districts and large cities not attached to an administrative district (termed district-free city in the following sections) with n = 401 (as of December 31, 2019) covering the whole of Germany [23]. A district is an association of municipalities and can be categorized into an urban district, a rural district with tendencies toward higher population density, and a sparsely populated rural district [23].

Information on physician density as an indicator of access to stroke care and treatment was identified from the German Federal Registry of Physicians [24]. Data on treatments in stroke units (code 8.981/8-98b in the German catalog of operations and procedures (OPS)) and recanalization therapy (systemic thrombolysis: OPS 8-020.8; mechanical thrombectomy: OPS 8-836.80) were obtained from the DRG data.

### Statistical analyses

Districts and district-free cities were categorized into quintiles of the GIMD 2015, with quintile 1 (Q1) comprising the least deprived (as reference) and quintile 5 (Q5) the most deprived areas. The stroke incidence rate was calculated age-standardized to the standard European population [25]. In the absence of age- and sex-specific mortality data at the district level, the age-standardized mortality rate was estimated by multiplying the age-standardized stroke incidence rate by the ratio of the total number of deceased to the total number of incident cases in the respective district, assuming that the proportion of deaths was the same among all age groups.

First, we performed univariate and bivariate analyses and created quintile-based choropleth maps to illustrate regional distributions. We then estimated rate ratios separately for ischemic stroke (IRR, incidence rate ratio) and mortality (MRR, mortality rate ratio) to explore GIMD 2015 differences by fitting negative binomial regression models, adjusting for the calendar year, age, sex, federal state, and district type. The regression equations are given in the appendix as Additional file 1. We fitted further linear regression models to assess the association between stroke care and treatment-related variables and the quintiles of the GIMD 2015, adjusting for federal state, and district type. We computed 95% confidence intervals (CIs), and the significance was set at *p* < 0.05. We did all analyses using STATA MP, version 17.

## Results

The population in Germany (83.17 million, as of December 31, 2019) is unevenly distributed among the 294 districts and 107 district-free cities. About 32.4% of the German population resides in a district-free city, and the majority of the population (67.7%) resides in a district. With a population of 3.67 million, Berlin has by far the largest population as a district-free city. In contrast, only 34,193 people live in Zweibrücken, the district-free city with the lowest population.

**Fig. 1:**
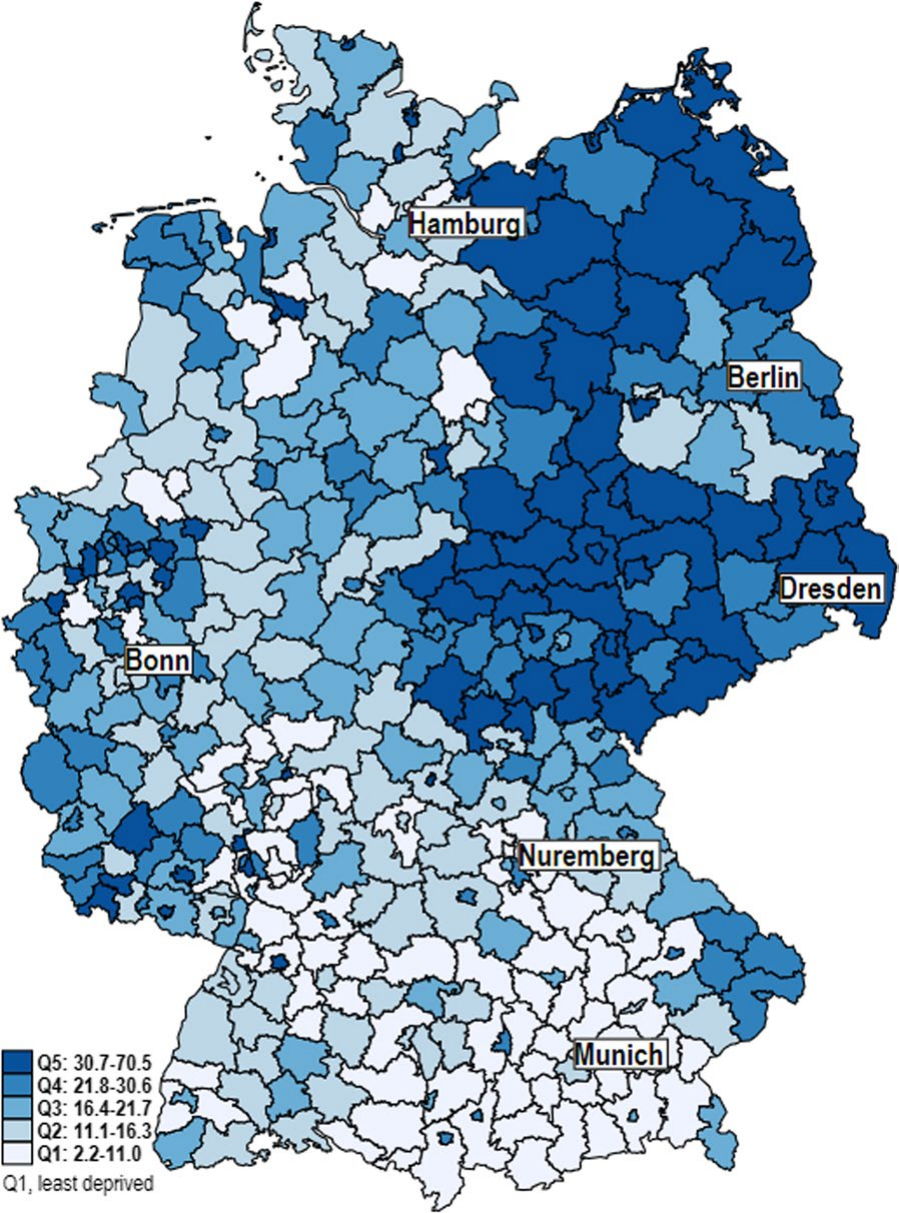
Distribution of the German Index of Multiple Deprivation (GIMD) based on districts and district-free cities in Germany

The GIMD 2015 total score varies from 2.2 (minimum) to 70.5 (maximum) across the n = 401 districts and district-free cities, and the arithmetic mean was 21.82. The Ebersberg district in Upper Bavaria has the lowest deprivation and the Mansfeld–Südharz district in Saxony–Anhalt has the highest. The distribution of deprivation quintiles is visualized in Fig. 1 and shows substantial regional differences. The districts in the eastern federal states show particularly high deprivation, whereas low deprivation is predominantly seen in the southern part of Germany. In the northwestern and southwestern federal states, regions with both high and low deprivation can be identified.

Moreover, the distribution of deprivation quintiles in Table 1 shows that more than half of the district-free cities have high levels of deprivation, while urban and rural districts tend to have lower levels.

**Table 1:** District types across GIMD 2015 quintiles (N = 401)

District types	GIMD 2015											
	Q1		Q2		Q3		Q4		Q5		Total	
	n	%	n	%	n	%	n	%	n	%	n	%
District-free large cities	4	6.0	9	13.4	11	16.4	19	28.4	24	35.8	67	100.0
Urban districts	47	35.9	34	26.0	28	21.4	12	9.1	10	7.6	131	100.0
Rural districts with tendencies towards higher population density	20	19.8	24	23.8	16	15.8	21	20.8	20	19.8	101	100.0
Sparsely populated rural districts	10	9.8	13	12.8	24	23.5	28	27.5	27	26.4	102	100.0

*n* quantity, % proportion, *Q1* first quintile, *Q2* second quintile, *Q3* third quintile, *Q4* fourth quintile, *Q5* fifth quintile

**Fig. 2:**
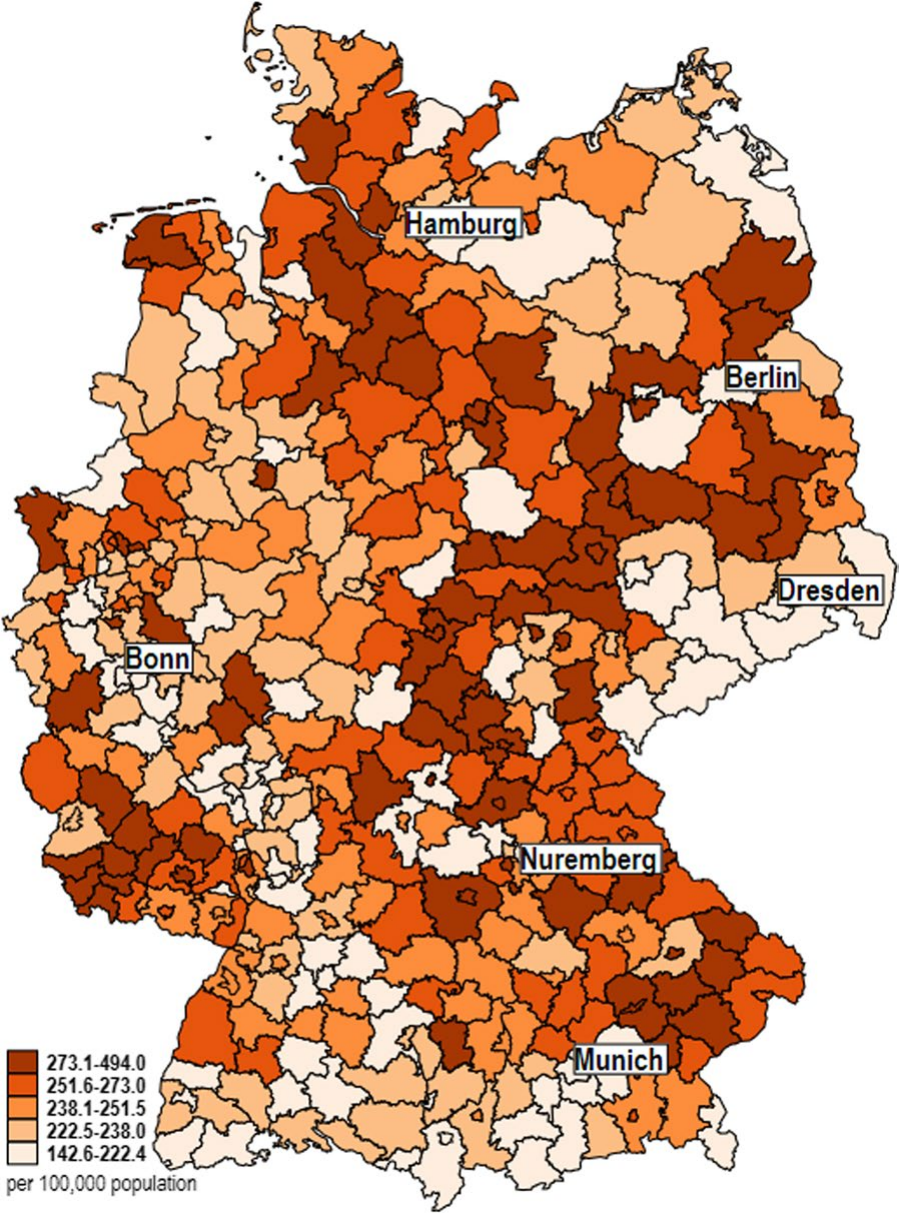
Distribution of the ischemic stroke rate of districts and district-free cities in Germany

The number of ischemic strokes treated in the hospital decreased by 0.95% between 2016 (n = 224,989) and 2019 (n = 222,841). The age-standardized rate (2016: 243.5; 2019: 236.1 per 100,000 population) decreased by 4.89% (men: − 2.75%; women: − 5.99%). Across the 401 districts and district-free cities, age-standardized stroke rates (average 2016–2019) ranged from 142 to 494 (men: 171–615; women: 113–405) per 100,000 population. Figure 2 illustrates the distribution of the ischemic stroke rates and again shows substantial regional differences. The districts and district-free cities along and south of the Elbe River (from Hamburg to Dresden), as well as Saarland and the eastern part of Bavaria (from Nuremberg to Munich), have particularly high incidence rates, while low rates can be found in the Ore Mountains (below Dresden) and the Alpine foothills (below Munich, border with Austria and Switzerland).

**Fig. 3:**
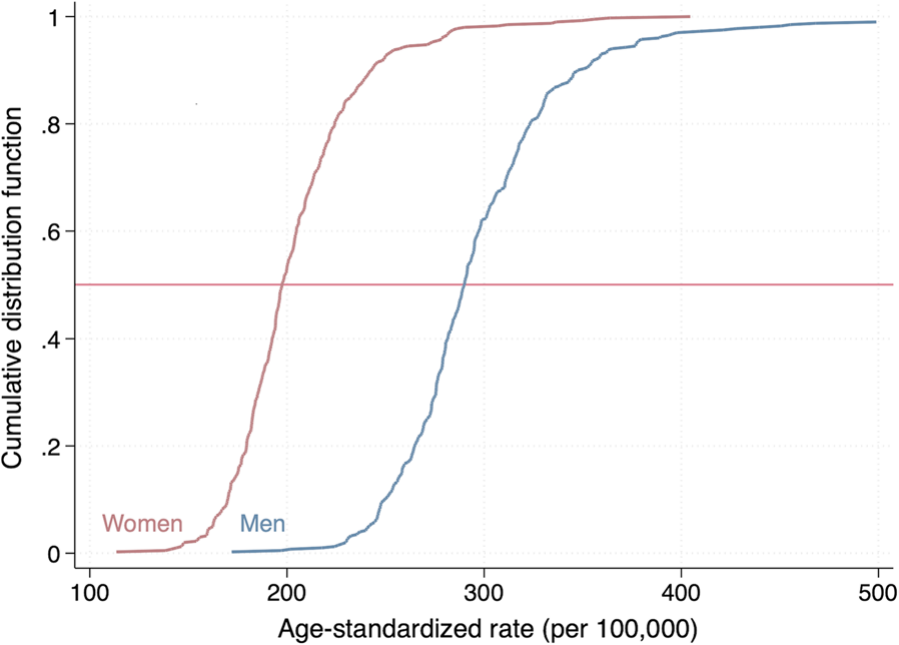
Cumulative distribution function of age-standardized stroke rates by sex

Figure 3 additionally shows the cumulative distribution function by age-standardized stroke rates for men and women separately, indicating that the median rate in women (197.6 per 100,000) is about 32% lower than the median rate in men (290.0 per 100,000).

Adjusted negative binomial regression between ischemic stroke incidence as the dependent variable and the GIMD 2015 as the independent variable showed an increase in ischemic stroke rate with higher levels of deprivation among the districts and district-free cities (upper half of Table 2), which is in line with our hypothesis.

**Table 2:** Results of negative binomial regression analyses on GIMD 2015 for stroke incidence and mortality (period 2016–2019)

Stroke incidence	Model 1.1^#^			Model 1.2^†^			Model 1.3^ǂ^		
	IRR	95% CI		IRR	95% CI		IRR	95% CI	
		Lower	Upper		Lower	Upper		Lower	Upper
*GIMD 2015*									
Q1, least deprived	1.0			1.0			1.0		
Q2	1.026	1.015	1.036	1.027	1.016	1.038	1.024	1.013	1.035
Q3	1.064	1.053	1.076	1.063	1.051	1.075	1.061	1.049	1.073
Q4	1.071	1.060	1.083	1.084	1.071	1.097	1.093	1.079	1.107
Q5, most deprived	1.114	1.102	1.126	1.138	1.122	1.154	1.161	1.143	1.179

Stroke mortality	Model 2.1^#^			Model 2.2^†^			Model 2.3^ǂ^		
	MRR	95% CI		MRR	95% CI		MRR	95% CI	
		Lower	Upper		Lower	Upper		Lower	Upper
*GIMD 2015*									
Q1, least deprived	1.0			1.0			1.0		
Q2	1.011	0.986	1.036	0.998	0.972	1.024	1.006	0.980	1.034
Q3	1.049	1.022	1.076	1.051	1.022	1.081	1.064	1.033	1.095
Q4	1.102	1.075	1.129	1.083	1.052	1.115	1.110	1.075	1.146
Q5, most deprived	1.167	1.138	1.197	1.153	1.114	1.193	1.193	1.148	1.239

*IRR* incidence rate ratio, *MRR* mortality rate ratio, *CI* confidence interval, *Q1* first quintile, *Q2* second quintile, *Q3* third quintile, *Q4* fourth quintile, *Q5* fifth quintile

^#^adjusted for year, age, and sex; ^†^adjusted for year, age, sex, and federal state; ^ǂ^adjusted for year, age, sex, federal state, and district type

Interestingly, physician density per 100,000 population was higher in Q4 and Q5 as compared to the lower GIMD 2015 quintiles (Table 3). No relevant differences were found with regard to admission to a stroke unit or treatment (see appendix, Additional file 2). There was only a significantly lower rate of mechanical thrombectomy in Q3 as compared to Q1.

**Table 3:** Stroke care and treatment-related variables across GIMD 2015 quintiles (N = 401)

	GIMD 2015				
	Q1	Q2	Q3	Q4	Q5
*Physician density (per 100,000)*					
Mean (SD)	156.5 (51.4)	162.7 (51.4)	173.8 (61.1)	186.9 (56.9)	180.0 (43.4)
Median	147.7	146.1	147.8	157.2	163.5
Q1, Q3	133.7, 169.4	136.4, 163.5	136.3, 201.9	142.6, 239.2	147.6, 218.7
Min, Max	87.1, 396.6	80.6, 345.1	90.6, 394.1	121.2, 314.8	104.2, 286.3
n	81	80	79	80	81
*Stroke unit care rate*					
Mean (SD)	0.724 (0.142)	0.747 (0.102)	0.739 (0.121)	0.726 (0.133)	0.729 (0.115)
Median	0.767	0.757	0.755	0.755	0.755
Q1, Q3	0.702, 0.803	0.700, 0.809	0.697, 0.816	0.684, 0.811	0.666, 0.801
Min, Max	0.188, 0.913	0.198, 0.966	0.315, 0.956	0.300, 0.899	0.362, 0.960
n	81	80	79	80	81
*Mechanical thrombectomy rate*					
Mean (SD)	0.070 (0.022)	0.071 (0.023)	0.062 (0.024)	0.070 (0.026)	0.067 (0.026)
Median	0.066	0.070	0.064	0.066	0.067
Q1, Q3	0.057, 0.082	0.054, 0.086	0.044, 0.076	0 .053, 0.085	0.046, 0.086
Min, Max	0.020, 0.152	0.027, 0.130	0.024, 0.151	0.014, 0.149	0.015, 0.135
n	81	80	79	80	81
*Intravenous thrombolysis rate*					
Mean (SD)	0.173 (0.043)	0.168 (0.040)	0.167 (0.039)	0.163 (0.046)	0.151 (0.043)
Median	0.166	0.164	0.166	0.159	0.147
Q1, Q3	0.138, 0.201	0.140, 0.190	0.139, 0.189	0.135, 0.186	0.123, 0.172
Min, Max	0.110, 0.314	0.054, 0.318	0.064, 0.291	0.075, 0.296	0.029, 0.320
n	81	80	79	80	81

*n* quantity, *SD* standard deviation, *Q1* first quintile, *Q2* second quintile, *Q3* third quintile, *Q4* fourth quintile, *Q5* fifth quintile, *Min* minimum, *Max* maximum

**Fig. 4:**
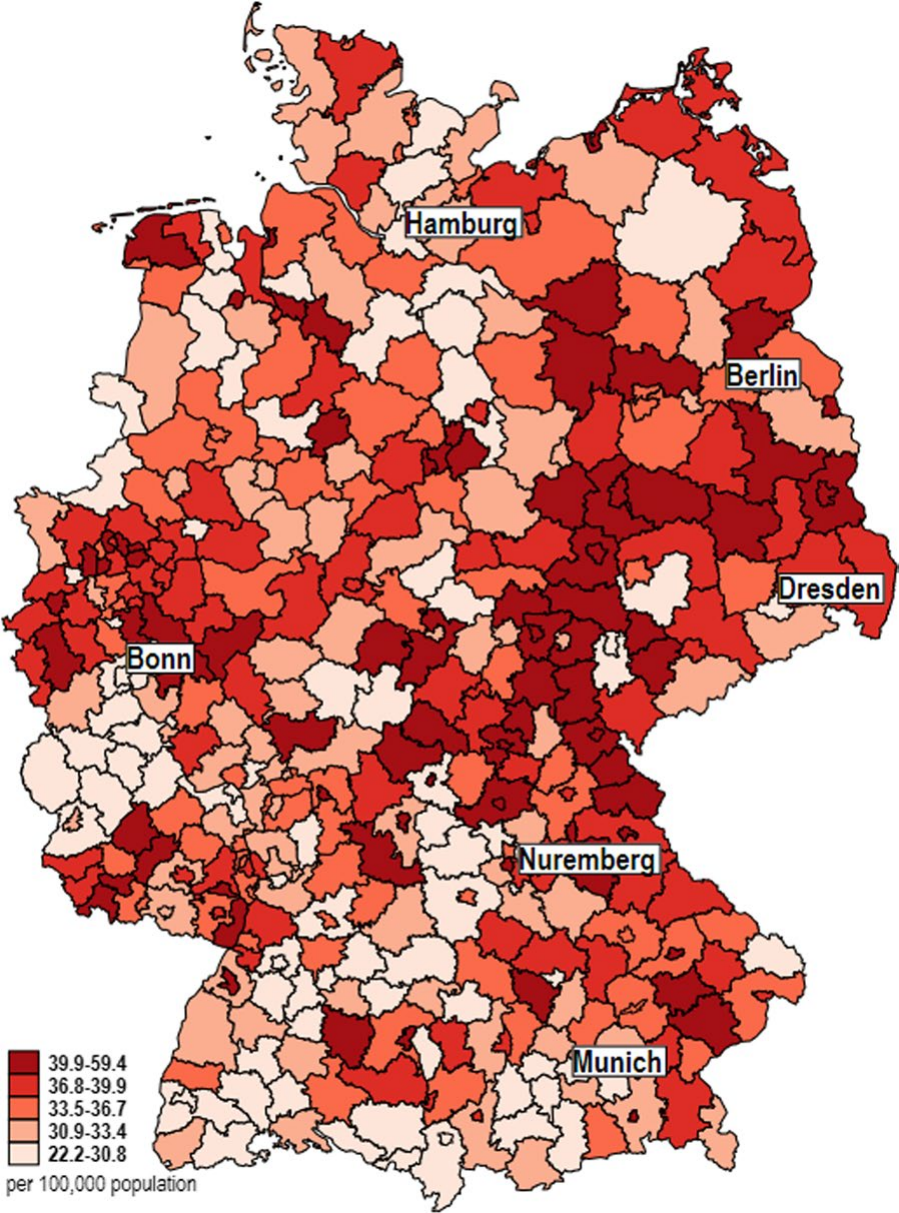
Distribution of the stroke mortality rate of districts and district-free cities in Germany

Considerable regional differences, however, are evident in stroke mortality (Fig. 4): age-standardized rates (average 2016–2019) ranged from 22 (minimum, Rottweil district in Baden-Wuerttemberg) to 59 (maximum, Hof district in Bavaria) per 100,000 population, and the arithmetic mean was 35 per 100,000 population. The number of deaths from stroke increased by 3.71% between 2016 (n = 15,996) and 2019 (n = 16,590). The age-standardized rate (2016: 17.5; 2019: 17.6 per 100,000 population) increased by 0.23%. It is noticeable that the minimum values of the mortality rates are mainly in Baden-Wuerttemberg, Rhineland-Palatinate, and Lower Saxony and the maximum values are predominantly in the eastern federal states.

The lower half of Table 2 displays the results of the negative binomial regression analyses between stroke mortality as the dependent variable and the GIMD 2015 as the independent variable showing an increase in stroke mortality with higher levels of deprivation among the districts and district-free cities, consistent with our hypothesis. Here, the observed associations between the GIMD 2015 and mortality are somewhat stronger than for the incidence of ischemic stroke. This indicates that survival after ischemic stroke is lower in more deprived regions.

## Discussion

This study examined the association between regional deprivation, ischemic stroke incidence, and mortality. We considered all documented hospitalized cases with the main diagnosis of ischemic stroke, and the effect of regional deprivation on key elements of stroke care and treatment. Our analyses show a statistically significant association between regional deprivation and hospital-treated ischemic stroke in Germany, which is comparable to other studies from different countries [12, 13, 26]. Moreover, our results demonstrate that the districts and district-free cities with the highest deprivation level have a 16% increased ischemic stroke rate compared to those with the lowest deprivation level. A registry-based cohort study of 44 million adults in England examining area-based socioeconomic disparities in the incidence of hospitalization for the first stroke found even more pronounced differences [13]. In our study, the effect size may have been underestimated because socioeconomic heterogeneity within the districts and district-free cities was not considered. Previous studies, however, found a statistically significant and independent association between regional deprivation and health outcomes after adjusting for individual SES [19, 27].

In addition to these findings, there was a decrease in the age-standardized incidence of ischemic stroke in the period from 2016 to 2019; a similar trend is found in other Western industrialized countries [28]. Conceivable explanations for the decreasing age-standardized incidence of stroke might be related to the lower prevalence of lifestyle risk factors such as tobacco and alcohol use [29] and public stroke awareness [30]. Although we did not find support for our hypothesis that the population in regions characterized by high levels of deprivation has poorer rates of stroke care and treatment, physician density was higher in districts and district-free cities that were more deprived. This could be explained by the fact that physician density is higher in district-free cities compared to districts. Although some studies showed an association between regional deprivation and worse intravenous thrombolysis rates [13, 31], studies indicating lower rates of mechanical thrombectomy in more deprived regions are not available.

Moreover, our results show a statistically significant association between regional deprivation and stroke mortality, which is also consistent with previous research [32–34]. Our results further demonstrate that the districts and district-free cities with the highest deprivation level have a 19% increased mortality rate of ischemic stroke compared to those with the lowest deprivation level. Similar effect sizes are observed for all-cause mortality [35]. A prospective cohort study in the U.S. assessing neighborhood mortality effects for people with versus people without a history of stroke reveals that neighborhood characteristics have similar estimated effects on stroke survival and individuals never having a stroke [32]. Additionally, age-standardized stroke mortality increased marginally from 2016 to 2019, contrary to the global trend [28].

Social deprivation can essentially be seen as a measure of "poverty", and districts and district-free cities with higher GIMD 2015 total scores may have less capacity to prevent and respond appropriately to ischemic stroke. A recent study shows that especially in the northeastern states, which we all classify as highly disadvantaged except Berlin, there is often no stroke care close to home, but rather delayed stroke care [36]. Therefore, the accessibility of stroke care in districts and district-free cities that are more deprived should be expanded, and telemedicine stroke networks can improve stroke care by providing access to time-dependent acute stroke treatment [36]. This can be complemented by a stroke awareness campaign and stroke prevention program in the affected regions.

The major strength of this study is its large sample size based on DRG statistics data of all hospitalized ischemic stroke patients resident in Germany. Limitations were that we could not consider individual-level SES as well as individual risk factors such as tobacco and alcohol use due to missing data. Further limitations include the joint consideration of first and recurrent ischemic stroke and the district level as the spatial unit of analysis. Further studies should attempt to compare spatial units that are as small as possible, e.g., at the neighborhood level. The districts considered here have a wide range in population size, and deprivation can be very heterogeneous within a district. When stroke and deprivation data are analyzed at a smaller scale, conclusions may change, which is known as the modifiable area unit problem [37]. However, since these data are not available for all of Germany, small-scale analyses are very difficult to perform.

## Conclusions

The population in regions in Germany characterized by high levels of deprivation has higher rates of incidence and mortality of hospitalized ischemic stroke cases, and physician density was higher in districts and district-free cities that were more deprived. Therefore, stroke prevention must focus more specifically on deprived regions.

## Supplementary Information

**Additional file 1**. Regression equations.

**Additional file 2.** Linear regression models between stroke care and treatment-related variables and the quintiles of the GIMD 2015.

## Data Availability

The datasets used and/or analyzed during the current study are available from the corresponding author on reasonable request.

## Abbreviations

CI: Confidence interval
DRG: Diagnosis related groups
GIMD: German Index of Multiple Deprivation
ICD-10-GM: International Statistical Classification of Diseases, 10th revision, German Modification
IRR: Incidence rate ratio
MRR: Mortality rate ratio
OPS: German catalog of operations and procedures
Q1: Quintile 1
Q5: Quintile 5
SES: Socioeconomic status
