# Supplementary material for: The impact of regional deprivation on stroke incidence, treatment, and mortality in Germany
Source: Neurol Res Pract. 2023 Feb 9;5:6. doi: 10.1186/s42466-023-00232-0 (PMC9909858; doi:10.1186/s42466-023-00232-0)
Supplement: Supplementary file 1 — Additional file 1. Regression equations. [file 42466_2023_232_MOESM1_ESM.docx]

1. Model for incidence

$\log\left( c_{ijkl} \right)=\log\left( n_{ijkl} \right)+\beta_{0}+ \beta_{1}sex+ \beta_{2}f\left( age \right)+\beta_{3}^{'} gimd+ \beta_{4} type$

1. Model for mortality

$\log\left( \hat{d}_{ijkl} \right)=\log\left( n_{ijkl} \right)+\beta_{0}+ \beta_{1}sex+ \beta_{2}f\left( age \right)+\beta_{3}^{'} gimd+ \beta_{4} type$

1. Model for letality

$\log\left( \hat{d}_{ijkl} \right)=\log\left( c_{ijkl} \right)+\beta_{0}+ \beta_{1}sex+ \beta_{2}f\left( age \right)+\beta_{3}^{'} gimd+ \beta_{4} type$

where

$c_{ijkl}$ - observed number of incident cases for age group *i*, sex *j*, year *k* and district *l*

$n_{ijkl}$ - observed population number in age group *i*, sex *j*, year *k* and district *l*

$\hat{d}_{ijkl}$ = $c_{ijkl}\times\frac{d_{. .kl}}{c_{..kl}}$ - estimated number of deaths in age group *i*, sex *j*, year *k* and district *l*

$d_{. .kl}$ - total number of deaths in year *k* and district *l*

$c_{. .kl}$ - total number of incident cases in year *k* and district *l*

*type -* 1 – urban, 0 - rural

*k* - 2017, 2018, 2019

*sex* - 0 – females, 1 – males

*age* - 5-year intervals 0-<5, 5-<10, …, 85-<90, 90+

Each district has assigned a value for GIMD15, which we categorized in quintiles. The first quintile (wealthiest) was used as baseline.

All estimates are adjusted for type of district (urban/rural)
