# Supplementary material for: The impact of regional deprivation on stroke incidence, treatment, and mortality in Germany
Source: Neurol Res Pract. 2023 Feb 9;5:6. doi: 10.1186/s42466-023-00232-0 (PMC9909858; doi:10.1186/s42466-023-00232-0)
Supplement: Supplementary file 2 — Additional file 2. Linear regression models between stroke care and treatment-related variables and the quintiles of the GIMD 2015. [file 42466_2023_232_MOESM2_ESM.docx]

**Table S1:** Linear regression model between physician density (as of 2019) as dependent variable and GIMD 2015

|  | |  | **Model S1.1^#^** | | |  | **Model S1.2**^†^ | | |  | **Model S1.3^ǂ^** | | |
| --- | --- | --- | --- | --- | --- | --- | --- | --- | --- | --- | --- | --- | --- |
|  |  |  | **IRR** | **95% CI** | |  | **IRR** | **95% CI** | |  | **IRR** | **95% CI** | |
|  |  |  |  | **Lower** | **Upper** |  |  | **Lower** | **Upper** |  |  | **Lower** | **Upper** |
| **GIMD 2015** | |  |  |  |  |  |  |  |  |  |  |  |  |
|  | Q1, least deprived |  | Ref. |  |  |  | Ref. |  |  |  | Ref. |  |  |
|  | Q2 |  | 6.15 | -9.99 | 22.28 |  | 13.20 | -3.22 | 29.62 |  | 13.80 | -0.33 | 27.93 |
|  | Q3 |  | 17.22 | 1.04 | 33.40 |  | 25.73 | 9.06 | 42.40 |  | 23.48 | 8.99 | 37.96 |
|  | Q4 |  | 30.41 | 14.28 | 46.54 |  | 46.89 | 29.34 | 64.45 |  | 36.14 | 20.15 | 52.14 |
|  | Q5, most deprived |  | 23.45 | 7.37 | 39.53 |  | 53.40 | 32.65 | 74.15 |  | 30.66 | 11.88 | 49.43 |
|  | *Intercept* |  | 156.50 | 145.20 | 167.90 |  | 142.05 | 123.00 | 161.10 |  | 121.50 | 105.10 | 137.80 |
| IRR, incidence rate ratio; CI, confidence interval; Q1, first Quintile; Q2, second quintile; Q3, third quintile, Q4, fourth quintile; Q5, fifth quintile; ^#^ unadjusted; ^†^ adjusted for federal state; ^ǂ^ adjusted for federal state, and district types | | | | | | | | | | | | | |

**Table S2:** Linear regression model between stroke unit care rate (as of 2019) as dependent variable and GIMD 2015

|  | |  | **Model S2.1^#^** | | |  | **Model S2.2**^†^ | | |  | **Model S2.3^ǂ^** | | |
| --- | --- | --- | --- | --- | --- | --- | --- | --- | --- | --- | --- | --- | --- |
|  |  |  | **IRR** | **95% CI** | |  | **IRR** | **95% CI** | |  | **IRR** | **95% CI** | |
|  |  |  |  | **Lower** | **Upper** |  |  | **Lower** | **Upper** |  |  | **Lower** | **Upper** |
| **GIMD 2015** | |  |  |  |  |  |  |  |  |  |  |  |  |
|  | Q1, least deprived |  | Ref. |  |  |  | Ref. |  |  |  | Ref. |  |  |
|  | Q2 |  | 0.023 | -0.016 | 0.061 |  | 0.004 | -0.034 | 0.042 |  | 0.014 | -0.024 | 0.053 |
|  | Q3 |  | 0.014 | -0.024 | 0.053 |  | 0.007 | -0.032 | 0.046 |  | 0.017 | -0.023 | 0.056 |
|  | Q4 |  | 0.001 | -0.037 | 0.040 |  | 0.002 | -0.039 | 0.043 |  | 0.015 | -0.029 | 0.058 |
|  | Q5, most deprived |  | 0.005 | -0.034 | 0.043 |  | 0.012 | -0.037 | 0.060 |  | 0.017 | -0.034 | 0.058 |
|  | *Intercept* |  | 0.724 | 0.697 | 0.751 |  | 0.771 | 0.726 | 0.815 |  | 0.759 | 0.714 | 0.804 |
| IRR, incidence rate ratio; CI, confidence interval; Q1, first Quintile; Q2, second quintile; Q3, third quintile, Q4, fourth quintile; Q5, fifth quintile; ^#^ unadjusted; ^†^ adjusted for federal state; ^ǂ^ adjusted for federal state, and district types | | | | | | | | | | | | | |

**Table S3:** Linear regression model between mechanical thrombectomy rate (as of 2019) as dependent variable and GIMD 2015

|  | |  | **Model S3.1^#^** | | |  | **Model S3.2**^†^ | | |  | **Model S3.3^ǂ^** | | |
| --- | --- | --- | --- | --- | --- | --- | --- | --- | --- | --- | --- | --- | --- |
|  |  |  | **IRR** | **95% CI** | |  | **IRR** | **95% CI** | |  | **IRR** | **95% CI** | |
|  |  |  |  | **Lower** | **Upper** |  |  | **Lower** | **Upper** |  |  | **Lower** | **Upper** |
| **GIMD 2015** | |  |  |  |  |  |  |  |  |  |  |  |  |
|  | Q1, least deprived |  | Ref. |  |  |  | Ref. |  |  |  | Ref. |  |  |
|  | Q2 |  | 0.001 | -0.007 | 0.008 |  | -0.002 | -0.010 | 0.005 |  | -0.002 | -0.009 | 0.005 |
|  | Q3 |  | -0.008 | -0.015 | -0.001 |  | -0.009 | -0.017 | -0.002 |  | -0.009 | -0.016 | -0.001 |
|  | Q4 |  | 0.000 | -0.007 | 0.008 |  | 0.001 | -0.007 | 0.009 |  | -0.001 | -0.009 | 0.007 |
|  | Q5, most deprived |  | -0.003 | -0.011 | 0.004 |  | 0.001 | -0.008 | 0.011 |  | -0.003 | -0.012 | 0.007 |
|  | *Intercept* |  | 0.070 | 0.065 | 0.075 |  | 0.087 | 0.078 | 0.095 |  | 0.081 | 0.073 | 0.089 |
| IRR, incidence rate ratio; CI, confidence interval; Q1, first Quintile; Q2, second quintile; Q3, third quintile, Q4, fourth quintile; Q5, fifth quintile; ^#^ unadjusted; ^†^ adjusted for federal state; ^ǂ^ adjusted for federal state, and district types | | | | | | | | | | | | | |

**Table S4:** Linear regression model between intravenous thrombolysis rate (as of 2019) as dependent variable and GIMD 2015

|  | |  | **Model S4.1^#^** | | |  | **Model S4.2**^†^ | | |  | **Model S4.3^ǂ^** | | |
| --- | --- | --- | --- | --- | --- | --- | --- | --- | --- | --- | --- | --- | --- |
|  |  |  | **IRR** | **95% CI** | |  | **IRR** | **95% CI** | |  | **IRR** | **95% CI** | |
|  |  |  |  | **Lower** | **Upper** |  |  | **Lower** | **Upper** |  |  | **Lower** | **Upper** |
| **GIMD 2015** | |  |  |  |  |  |  |  |  |  |  |  |  |
|  | Q1, least deprived |  | Ref. |  |  |  | Ref. |  |  |  | Ref. |  |  |
|  | Q2 |  | -0.005 | -0.018 | 0.008 |  | -0.004 | -0.017 | 0.009 |  | -0.003 | -0.017 | 0.011 |
|  | Q3 |  | -0.007 | -0.020 | 0.006 |  | -0.003 | -0.017 | 0.010 |  | -0.003 | -0.017 | 0.011 |
|  | Q4 |  | -0.011 | -0.024 | 0.002 |  | -0.003 | -0.017 | 0.011 |  | -0.003 | -0.019 | 0.012 |
|  | Q5, most deprived |  | -0.022 | -0.035 | -0.009 |  | -0.007 | -0.024 | 0.009 |  | -0.009 | -0.028 | 0.009 |
|  | *Intercept* |  | 0.173 | 0.164 | 0.182 |  | 0.183 | 0.168 | 0.198 |  | 0.180 | 0.164 | 0.196 |
| IRR, incidence rate ratio; CI, confidence interval; Q1, first Quintile; Q2, second quintile; Q3, third quintile, Q4, fourth quintile; Q5, fifth quintile; ^#^ unadjusted; ^†^ adjusted for federal state; ^ǂ^ adjusted for federal state, and district types | | | | | | | | | | | | | |
